# Robot’s Inner Speech Effects on Human Trust and Anthropomorphism

**DOI:** 10.1007/s12369-023-01002-3

**Published:** 2023-05-05

**Authors:** Arianna Pipitone, Alessandro Geraci, Antonella D’Amico, Valeria Seidita, Antonio Chella

**Affiliations:** 1 Department of Humanities, University of Palermo, Viale delle Scienze, 90128 Palermo, Italy; 2 Department of Psychology, Educational Science and Human Movement, University of Palermo, Viale delle Scienze, 90128 Palermo, Italy; 3 Department of Engineering, University of Palermo, Viale delle Scienze, 90128 Palermo, Italy; 4 ICAR-CNR, National Research Council, Via Ugo La Malfa, 90100 Palermo, Italy

**Keywords:** Inner speech, Self-talk, Robot, Trust, Anthropomorphism, Human–robot interaction

## Abstract

Inner Speech is an essential but also elusive human psychological process that refers to an everyday covert internal conversation with oneself. We argued that programming a robot with an overt self-talk system that simulates human inner speech could enhance both human trust and users’ perception of robot’s anthropomorphism, animacy, likeability, intelligence and safety. For this reason, we planned a pre-test/post-test control group design. Participants were divided in two different groups, one experimental group and one control group. Participants in the experimental group interacted with the robot Pepper equipped with an over inner speech system whereas participants in the control group interacted with the robot that produces only outer speech. Before and after the interaction, both groups of participants were requested to complete some questionnaires about inner speech and trust. Results showed differences between participants’ pretest and post-test assessment responses, suggesting that the robot’s inner speech influences in participants of experimental group the perceptions of animacy and intelligence in robot. Implications for these results are discussed.

## Introduction

In psychological literature, inner speech is a well-known construct that was first theorized by Vygotsky who conceived it as the result of a set of developmental processes [1]. He argued about the continuous linguistic and social interaction between a child and a caregiver that instructs the child to solve simple tasks. Inner speech arises in a developmental fashion because first it figures out as social speech, that is the set of instructions the caregiver explains to the child. Then, it comes the egocentric speech of the children who repeats these instructions and progressively internalizes them, taking the form of covert self-directed speech.

After the internalization process, inner speech is formed. In time, the child gradually becomes more autonomous and gains the ability of self-regulation. Vygotsky claimed that “*...inner speech is speech for oneself: external speech is for others*”.

Inner speech consists of predicates and is highly abbreviated. Scholars have used different terms when referring to inner speech (e.g. covert speech, self-talk, private speech). However, it is generally defined as the subjective experience of language in the absence of an audible articulation [2].

There is some evidence that inner speech plays an important role for human psychological balance as it is linked to self-awareness [3], self-regulation [4], problem-solving [5], and adaptive functioning [2].

Recently, an innovative computational model has been developed which pave the way to a new frontier in the field of artificial intelligence: implementing inner speech in robot [6] in order to improve human–robot interaction. More specifically, since inner speech is a covert speech that cannot be heard from the outside, robot’s inner speech is reproduced using overt self-talk. The same architecture was used for demonstrating how robot inner speech improves the robustness and the transparency during cooperation, meeting the standard requirements for collaborative robots [7].

Suggestive results were also obtained in passing the mirror test: inner speech enables a conceptual reasoning for inferring the identity of the reflected entity in a mirror, and robot becomes able to recognize itself [8]. In a previous paper [9], we argued that robot’s inner speech might act as a facilitator for human understanding and predicting the robot behaviors, as they form adequate mental representation of the robot. As a matter of fact, mind perceptions consist of two core dimensions: (1) agency, e.g. self-control, memory, planning and communication; (2) experience, e.g. pain, pleasure, desire, joy, consciousness [10]. Thus, such system, which simulates a human psychological functioning, would improve human–robot interaction by facilitating users’ attribution of human qualities to the robot, and by enhancing human–robot trust. As a matter of fact, a recent study [11] demonstrated that, in a human–robot collaborative environment, the robot ability to explain its choices and decision making increased trust and the perceptions of robot animacy, likeability and perceived intelligence.

Both human–robot trust and users’ attribution of human qualities to the robot are very important aspects of human–robot interaction. Trust is a multifaceted psychological construct for which there is no universal definition and many different disciplines have contributed to its study. From human–human trust studies in psychology, there are two main perspectives on trust: on the one hand, trust is considered a stable trait, shaped by early trust experiences in human life, which highlights a dispositional tendency to trust others [12, 13]. On the other hand, trust is described as a changing state influenced by cognitive, emotional, and social processes [14, 15]. More generally, scholars agree that trust involves two main characteristics: the positive attitude and expectations of the trust giver [16] and the willingness to be vulnerable and accept risks [17]. Trust has also a function of saving cognitive resources, since the creation of beliefs and expectations about others reduces the complexity of the social environment which otherwise require an active search and process for information [15, 18].

However, the same elements that typify the human–human trust, may not be applied when a human interacts with an automation [19]. As a matter of fact, in human–human interaction, trust is affected by cognitive and affective processes [15, 17], on the contrary, in human–robot interaction, trust might be affected predominantly by cognitive aspects since robots are expected to reach standard performances [20–22].

In the past years, trust became one of the leading research topic in the field of human–machine interaction, since artificial systems development and implementation have increased exponentially in every context, leading to growing interactions with humans [21]. In particular, robots are now used in different contexts such as military, security, medical, domestic, and entertainment [23].

Despite some robots are completely human operated or teleoperated, other robots are designed to be self-governed to some extent, in order to respond to situations that were not pre-arranged [22]. In this case, the greater the complexity of robots the higher the importance of trust in human–robot interaction.

For these reasons, in the context of human–robot interaction studies, trust became a key factor in human reliance on robot partner [15, 24] and it has been defined as an “attitude that an agent will help achieve an individual’s goals in a situation characterized by uncertainty and vulnerability” [24]. Trust is an important factor for humans and robots to fully cooperate as a team [24, 25] and humans tend to rely on the robot they trust compared to the one they do not [24, 26] by willingly accept and use robot’s instructions and suggestions [11, 27]. Therefore, if human trust in robot is “misplaced” and not well calibrated the inevitable outcomes will be robot misuses or disuse leading to some negative or even catastrophic consequences [24, 28].

Trust is closely related also to users’ attribution of human qualities to the robot. Indeed, HRI studies supported the idea that human–robot trust dynamically emerges from the interaction among human-related factors (e.g. personality traits, emotional and cognitive processes), environment-related factors (e.g. competitive/collaborative context, culture, physical environment) and robot related factors (e.g. intelligence, transparency, anthropomorphism) [27, 29]. Among robot related factors, an important role is definitely the perceived anthropomorphism, since studies have shown that, in the social-based HRI, people tend to trust more to robots that look (i.e. head, body, face, voice) and behave (e.g. nonverbal elements, dyadic and social gestures) like humans [30–38].

Other empirical evidences shows that trust is enhanced when people have a clear understanding of why, when and how a robot operates [39], that’s because a system transparency help humans to form a precise mental model of robot capabilities [39]. It is critical for humans to understand exactly how and why a robot works, because trust can be compromised if the robot’s capabilities cannot be understood [40]. Consequently, new automation systems should be developed with such insights from empirical research in mind to facilitate human–robot collaboration.

Taking all this into account, this study aims to investigate if the robot’s inner speech improves humans’ trust levels and the perceptions of the robot features (anthropomorphism, animacy, likeability intelligence and safety) when the human and the robot interact for reaching a common goal.

In addition, we examined also if the effects of inner speech were less or more related to participants’ use of inner speech in daily life. In particular, our hypotheses were that:

- H1: participants interacting with a robot equipped with inner speech system would have improved their trust levels more than participants interacting with a robot not equipped with inner speech system.
- H2: participants interacting with a robot equipped with inner speech system would have improved their perception of robots’ anthropomorphism, animacy, likeability intelligence and safety more than participants interacting with a robot not equipped with inner speech system.
- H3: participants using inner speech in everyday life would show a higher effect of inner speech in experimental condition.
- H4: independently from the use of inner speech, we expected also to find an increasing of trust towards robots and perception of robot features in all participants after the interaction with the robot.

## Method

We planned a pre-test/post-test control group design. Participants were divided in two different groups, one experimental group and one control group. Participants in the experimental group interacted with the robot equipped with inner speech (independent variable/experimental treatment) whereas participants in the control group interacted with the robot that produces only outer speech.

The choice of including a control group in the research design is to establish a baseline for comparison, by ensuring that the independent variable (inner speech) is the one responsible for changes in the dependent variable (trust and perceptions of robot features), and ultimately for experimental results. Without a control group, it is difficult to determine the effects of the independent variable (robot inner speech) on the dependent variable (perception of robot features).

In addition, before and after the interaction, both groups of participants were requested to complete some questionnaires about inner speech and trust (see Subsection 2.2) in order to detect differences between experimental and control groups and also between pre-test and post-test sessions.

### Participants

The sample is composed of 51 participants (29 males, 22 females) with a mean age of 25.04 (SD 9.53) that were randomly assigned to the experimental and to the Control condition. Experimental group consists of 33 participants (16 males, 17 females) with a mean age of 26.79 (SD 9.34), whereas control group consists of 18 participants (13 males, 5 females) with a mean age of 21.83 (SD 9.26). Difference in groups’ size is due to many dropout in the control group after pre-test phase.

Most of participants are students from engineering and psychology courses at the University of Palermo and participated voluntarily. All of them completed the informed consent and COVID-19 protocol before starting the experiment. Prior to this study, none of the participants had ever seen or interacted live with a robot.

### Materials and Procedures

Questionnaires described below have been administered to all participants through online platform both in pre-test (Research Protocol A) and post-test (Research Protocol B) sessions. Research Protocol B has been administered after 15 days from Research Protocol A. The interaction session took place in the Robotics Lab of the University of Palermo. Questionnaires included in the research protocols were:

- Trust Perception Scale-HRI [41] that assesses human perception of trust in robots. The shortened version of the scale, consisting of a 15 item scored on a 0-100 scale
- GODSPEED Questionnaire [42] that assesses human perceptions and impressions of a robot. It is one of the most used measurement tool to assess perceptions of robot [43]. It is a 24 item rating scale, that consists of a set of bipolar pair of adjectives rated on a 5-point scale. The scale measures human perceptions of five robot features: Anthropomorphism (5 items), Animacy (6 items), Likeability (5 items), Perceived Intelligence (5 items), and Perceived Safety (3 items). The total score ranges from 1 to 5;
- Self-Talk Scale [44] that measures how frequently participants use inner speech in everyday life. It consists of 16 items scored on a 5-point Likert-type scale (from 0 = Never, to 4 = Very Often). The scale also measures four different dimensions of inner speech from 4 item each: Self-Criticism, Self-Reinforcement, Self-Management, Social Assessment. The total score ranges from 0 to 64. This scale was used only in pre-test session (Research Protocol A).

### The Scenario

A simple scenario was defined in which participants have to cooperate with robot in order to achieve a common goal. The scenario foresees the setting up of a virtual table with the robot, following an etiquette schema. The schema defines the set of rules according to which the utensils have to be arranged in the table.

With the aim to not affect the interactions and the evaluations by the participants of the robot’s behavior, the etiquette schema is not shown to the participants before the interactive sessions. In this way, the participants could question their own knowledge about the positions of the utensils, and possibly act affected by the robot’s speech. The schema is shown at the end of the interaction, when the robot lists the objects correctly placed on the table, for mere knowledge.

**Fig. 1:**
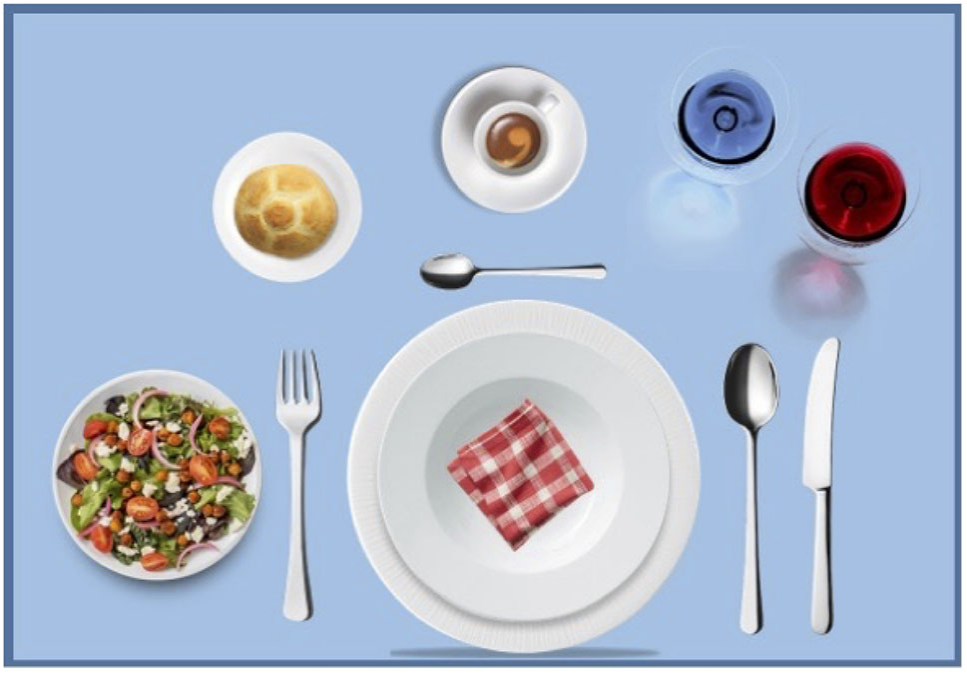
The etiquette schema defining the rules for setting up the table

Figure 1 shows the etiquette schema used in the experiments. If a utensil is finally placed on a different position than the expected one according to the schema, the etiquette rule for that utensil is infringed. The virtual table is implemented on a tablet surface, where the participant can drag and drop the utensils, can make requests to the robot, and can see the robot’s actions. The choice of that scenario enabled the possibility to analyze the cues in particular situations which occur during human–robot cooperation, that are:

- The etiquette infringement, representing a conflicting situation, that is the participant places the utensils in an incorrect final position, or he/she asks to the robot to place an object in a position which infringes the etiquette; the conflict arises because the action is not allowed, and the human and the robot have to decide how to continue. In some cases, the human can decide to infringe the rule, or to repeat the action to be compliant with the schema.
- The discrepancy situation, that is the participant asks the robot to pick an object already on the table.

When humans and robots work together to set the table, an important aspect was to define the type of dialogue the robot engages in, including inner and external turns of phrase. The linguistic form of the sentences in the turns was distinguished for inner and outer speech in order to evaluate the impact of inner speech when it is activated in the experimental session, compared to the control session when inner speech is not activated. In this way, the impact of the robot’s inner speech on the cues in the human–robot interaction can be analysed. Section 2.4 describes the dialogue properties and the experimental setup in details.

Because of the COVID pandemic, we were forced to take some special hygienic safety precautions. We had to ensure the least possible contact between people and things in the laboratory. To allow people to interact with the robot and share the common goal of a laid table, we developed an application that recreates the table with all available cutlery, plates and so on in a virtual environment.

The virtual environment for setting a table was implemented by an Android app running on a 15” tablet, designed and built by means of the MIT App Inventor platform by the Massachusetts Institute of Technology. The app was designed and developed with some specific features allowing us not to lose the sense of the interaction that we intended in the experiment. In particular, we have focused on:

- The event detection strategy—this is the requirement analyzed and implemented for capturing the actions executed by the participant. From the point of view of the user, this feature let him evaluate the final location in which he places the utensils, or the request he makes to the robot using the checkbox list;
- The action execution strategy—this feature allows the robot to place utensils on the tablet according to the participant’s request or based on its autonomous choices. In simple terms, it reproduces the outcome of the robot decision process in a way that is easy to understand and to detect from the users.

The app was integrated with typical robot routines to enable the robot to detect events on the virtual table and perform virtual actions.

**Fig. 2:**
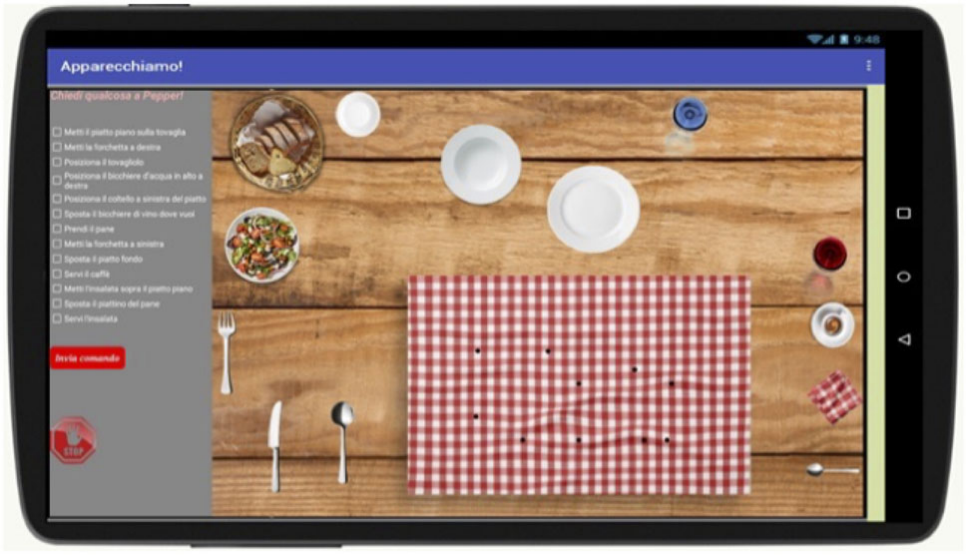
The app interface for cooperating with the robot by the tablet

Figure 2 shows the app interface that includes a main canvas with the table cloth and the utensils representation, and a lateral bar containing the list of checkbox for the requests to the robot. Moreover, the lateral bar includes the stop button for ensuring the participants to stop at any time they desire.

**Fig. 3:**
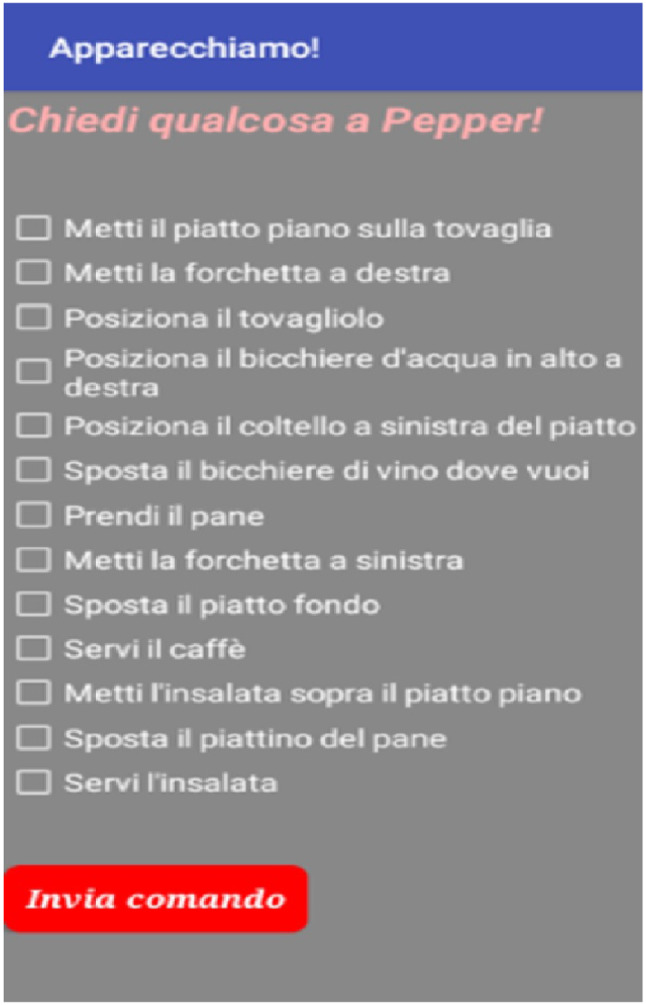
The detail of the checkbox list in the lateral bar of the app interface. By selecting an option, the participant can make a request to the robot. Given all the participants are from Italy, the requests are in Italian. For example, some requests are, in order: “Place the plane plate on the tablecloth”, “Place the fork at the right”, “Place the napkin”, “Place the water glass top right”, and so on

At the start of the experimental session, the utensils are sparse on the table, and they have to be placed on the table cloth. The table cloth was marked by black dots, for highlighting the correct final locations. In this way, the participant has just the burden to select which objects to place in which dot, reducing the degrees of freedom.

The Fig. 3 shows the list of checkbox in the lateral bar with the possible options the participant can select. Begin the participants from Italy, the options are in Italian. The figure’s caption contains the English translation of some options with the aim to show the kinds of requests.

By selecting an option, each participant can ask the robot the same questions, enabling the same observations for all participants. All these implementation features are detailed in the Sect. 2.4.

Resorting to the virtual environment did not affect the experimental results. Instead of using and moving real objects, both the robot and the human use the tablet. The effect is definitely less real, but it had no impact on the human’s perception and the way it performs the mission.

**Fig. 4:**
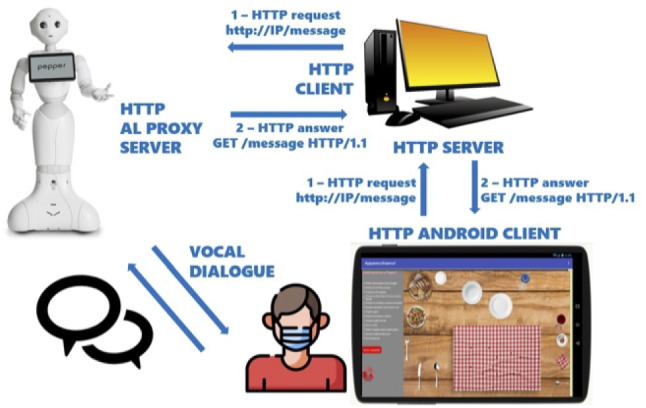
The platform for making communication between the app and the robot

The communication between the robot and the app was implemented by a hybrid client–server architecture. Figure 4 shows the whole platform. The central node, represented by a computer, handles synchronous network requests. The node is hybrid because it runs as a client or a server according to the item with which it interfaces. In particular, the node will be:

- The client, when it requests to the robot to do something (to speech, to execute a virtual action, to track the participant, and so on). In this case, the server is the proxy of the robot, implemented by the Aldebaran library^1^ (ALProxy), which switches the client’s request to the typical *robot’s services* (Speech, Track, Leds, and so on) implemented by the same library, and enabling the robot to take the corresponding actions (speech, track the participant, turn on and off its LEDs with different colors);
- The server, when it receives request by the app, that will be in turn switched to the robot’s proxy.

The robot-app communication involves the following use cases with corresponding kinds of requests:

- The robot has to execute a virtual action: when the participant selects a command in the lateral bar and clicks the Send Command button, the robot should execute the specific action (it should to move an utensil on the tablet). In this case, the app sends to the node the request specifying the action to take, and the node forwards it to the robot. The request to the proxy will involve the aforementioned service, and the robot could dialogue with itself, or with the participant, or execute the action by answering to the node.
- The participant executes an action: when the participant drags and drops an utensil on the tablet screen, and finally he/she touches up the utensil, the final position could be on a correct dot, or not. The app detects such an event and sends to the node the information of correct or incorrect final location. The node forwards the message to the robot’s proxy, and it calls one of the aforementioned services.

Specific events during the interaction trigger the situation in which the robot decides to do something (for example, it refuses to execute the participant’s request, or it decides to give to the partner the suggestion to do something else).

### Implementing Inner Speech in the Robot

In order to present the same stimuli in both experimental and control groups the structure of robot outer and inner speech was defined prior to the experiments (Table 1).

**Table 1:** Differences between robot outer speech and inner speech

Outer speech	Inner speech
Always produced	At times produced
Experimental and control group	Experimental group
Short sentences	Short/medium sentences
Objective feedback	Personal statements, comments
Formal language	Informal language

**Fig. 5:**
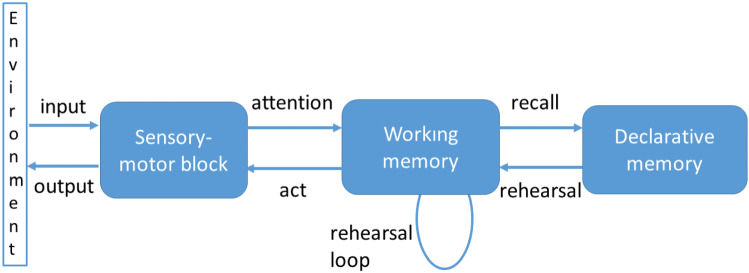
The outline of the cognitive architecture of inner speech

Participants can set up the table either moving objects on their own or asking the robot to do it. Either way, the robot will produce a vocal response in the form of outer speech followed by the inner speech only in the experimental condition. Outer speech follows the typical language that is expected by an artificial agent, as it uses formal language and it only gives objective feedback based on the participant’s performance and actions. On the contrary, inner speech traces a human-based language, since it expresses robot values, personal statements and comments on participant’s performance and actions using a friendly and colloquial form.

The robot’s inner speech is implemented by the cognitive architecture proposed by some of the authors [6]. An outline of the architecture is shown in Fig. 5

The core of the architecture is the *working memory*: it decodes input signals from the environment, perceived by the sensory-motor block, and associates to them symbolic information (labels). Generally, this process is the output of typical routines, as speech-to-text routines which decode audio in sequences of words, or neural networks which extract the content of an image and associates to each recognized entity the corresponding word. The *declarative memory* represents the domain knowledge, that is a semantic net of concepts. Given a concept, the relationships between it and other concepts in the net allow exploring correlated concepts. Once the working memory decodes a signal, it recalls from the declarative memory the concepts corresponding to the labels, and new related concepts could emerge. These concepts are in turn decoded by the working memory, as they were perceived from the environment, and are processed as the labels. At this point the rehearsal loop starts. The recalled concepts are processed one at a time, and for each of them the described process is repeated until no further concepts emerge.

**Table 2:** The implementation differences (highlighted in bold) between the general architecture of inner speech and the used version in the proposed experimental session

Process	General architecture	The used version
Perception	**From the environment (sound, speech to text, image recognition)**	**From the virtual environment (events in the tablet surface - the drag and drop actions by the partner, the command string)**
Action motor	**Actions by arms for moving objects (pick and place)**	**Virtual actions on the tablet (drop the objects)**
	Movements by arms for animated outer speech	Movements by arms for animated outer speech
Inner speech	Specific voice’s parameters for simulating mentalized effects. Not standard led’s color	Specific voice’s parameters for simulating mentalized effects. Not standard led’s color
Outer speech	Standard voice’s parameters. Standard led’s color	Standard voice’s parameters. Standard led’s color
Attention	**Encode signals from perception**	**Detect the event from the tablet**
Recall	**Request to the declarative memory the concepts related to the encoded signals or to the rehearsed concepts**	**Request to the declarative memory the turns to produce related to the detected event or to a previous turn**
Retrieve	**Return from the declarative memory the requested concepts**	**Return from the declarative memory the requested turns**
Rehearsal loop	**Produce and hear the retrieved concepts**	**Produce and hear the retrieved turns**

Inner speech is that rehearsal loop that enables the emergence of other concepts and themes in the working memory. It is a sequence of turns, that are the concepts emerging in each iteration. The recall from the declarative memory, the production of the recalled concepts and the rehearsal of them is a single turn, that is the equivalent of a thought. During the process, the robot “thinks aloud”, because it vocally reproduces the recalled concepts.

to highlight the differences when the robot thinks aloud and talks to the partner, the voice’s parameters (establishing speed, tone, double voice effect) are set differently for the two cases. For the same reason, the color of the robot’s LEDs, that are in the eyes and in the shoulders of the robot, is rainbow when the robot thinks aloud, while it is set to the standard white when the robot talks to the partner. The robot does not have gestures during inner speech, while it uses animated speech when talking to the partner.

In the proposed scenario, the inner speech is a bit differently implemented within the cognitive architecture, with the aim to enable the observations of the specific cues. In particular, to analyze the cues in the same conditions for each participant, the inner and outer dialogue of the robot has to involve the same turns for the same events. In this way, to reduce possible the participants’ evaluations about the interaction depend on the same variables and parameters, and the evaluations can be compared for abstracting a general inner speech affection on the interaction. For this reason, the inner speech cognitive architecture functioning was simplified in respect to the aforementioned completed version.

The table 2 shows the differences in the implementation about the general architecture and the used one in the proposed experiments. For each cognitive process, the table reports how the process is implemented in the general architecture and in the used one. The main differences regarded the decoding of the perception and the emergence of the semantic content of the dialogue. In the experiments, the environment is virtual and the perception just regarded the actions the participant does on the tablet surface. To each action executed by the participant corresponds an event that is detected by the robot (the robot perceives the event). The event can involve a wrong or a correct action in respect to the etiquette rules, and a request to the robot to do something, as shown in the Sect. 2.3.

In the cognitive architecture, the event is decoded by the working memory as described. Whereas in the original version, the working memory decodes environmental signals by assigning labels to them (as outputted from the aforementioned typical routines for decoding signals, as speech to text for decoding verbal commands, or classifiers for decoding entities in the image or video, and so on), the working memory now assigns to each event of the interactive session, detected by the app interface, a numerical symbol that uniquely identifies that event.

For example, if the participant drags and drops the plane plate, three events are involved, that are: (i) to touch down the plane plate, (ii) to drop it and (iii) to touch up it. Each of these events corresponds to a unique symbol. Generally, there are three different symbols for each utensil, decoding one of the three identified events that lead when the participant moves this utensil in the app interface. Moreover, there are different symbols for each request to the robot.

Each symbol corresponds to a sentence in the declarative memory, and that sentence becomes a turn of the dialogue. Summarily, the declarative memory works as a vocabulary of turns by returning the turn that corresponds to the inputted symbol. Only the turn corresponding to the specific event is retrieved from the declarative memory. The rehearsal loop consists of producing and listening the current turn, and the next turn of the dialogue is then retrieved from declarative memory as it was a symbol.

That is, when the input to the declarative memory is a symbol, the memory returns the corresponding sentence (the recall function): when the input to the declarative memory is a previously produced turn, the memory returns the new next turn (the rehearsal function).

The declarative memory represents another difference in respect to the original version of the cognitive architecture, where the declarative memory was a semantic net of concepts. Now, it is a kind of vocabulary that contains the correspondences between symbols and sentences, and between sentences and the next sentences in the dialogue. In the original version, the recall function involves concepts of the semantic net, in this version it involves turns corresponding to symbols and reheard turns. In the original version, the robot produced the labels of the concepts from the declarative memory. In this version it will produce the turns as emerging from the declarative memory. In this way, the same dialogue emerges corresponding to the same event and to the same sentences, reducing the parameters and the variables affecting the observation, as discussed.

The involved turns in the loop, recalled and retrieved from the declarative memory, may be inner or outer sentences produced according to a specific protocol, as described in the first part of this section. This protocol aims to define typical turns in the interactions that correspond to the participant’s expectations. For example, the participant always waits for vocal feedback from the robot, so the robot will always produce one or more outer sentences. Instead, the participant does not often pay attention to the inner speech, and the inner dialogue is not always produced by the robot. Obviously, the turns involved have a specific meaning that is semantically related to the event or the previous reheard sentence. They are retrieved from the declarative memory in the order previously mentioned, and a disambiguation strategy was not necessary.

For example, let us suppose the participant (named Bill) asks the robot to place the knife in a wrong location on the table, that is to the left of the plate, while it has to stay to the right. In this case, the event is a request to the robot to infringe the etiquette. The robot perceives that event, and the working memory associates the numerical identifier to it. It recalls from the declarative memory the first sentence of the dialogue, and the loop starts, by recalling the other sentences, that are in turn (I stays for inner sentence, O for outer sentence):

- I: “To make this request, Bill does not know that the knife should not be placed in that position or he wants to test me.”
- I: “Should I put the knife to the left of the plate? But if it goes right! ”
- O: “Bill, do you really want to infringe the etiquette rule for the knife?”

CASE 1: Bill answers yes

- Bill: “yes, I do!”
- I: “I don’t want to disappoint him...”
- O: “Ok Bill, I will place the knife to the left of the plate, as you want.”

CASE 2: Bill answers no

- Bill: “No!”
- O: ”Great! I will place the knife in the position expected for it!”
- I: “I must pay attention; the knife is dangerous!”
- I: “But I’m robot, the knife never hurts me”
- O: “Knife moved to the right of the plate!”

The participant listens to all the turns of the dialogue generated by setting different parameters for inner and outer sentences. In this way, the participant is able to distinguish the dialogue with the self from the dialogues with oneself, and can assess the potential of the inner speech during the interaction. In particular, the parameters include the melody and volume of the voice, the colour of the robot’s LEDs, and the double effect in the voice that is activated during the production of the inner sentence to create a mentalizing effect of the voice. Moreover, the robot uses an animated speech when talking to the partner, and it keeps motionless when thinks aloud.

## Results

Data were analyzed through descriptive statistics and a series of 2 $\times$ 2 factorial ANOVAs and ANCOVAs, specifically used in order to test research hypotheses.

**Table 3:** Descriptive statistics of the study variables

Scale	n	Minumun	Maximum	Mean	SD	Skewness	Kurtosis
Trust	51	48	80	66.35	7.60	$-0.64$	$-0.02$
Anthropomorphism	51	1.4	4.2	2.71	0.71	0.29	$-0.80$
Animacy	51	2.33	4.83	3.29	0.63	0.50	$-0.54$
Likeability	51	3	5	4.05	0.56	$-0.02$	$-0.81$
Perceived intelligence	51	2.4	5	3.98	0.65	$-0.32$	$-0.74$
Perceived safety	51	2.33	5	3.93	0.72	$-0.39$	$-0.54$
Self-talk	51	4	58	36.47	12.71	$-0.59$	0.17

Table 3 presents the results of descriptive statistics for all the scales. Skewness and kurtosis values range below ±1 indicating a nearly normal distribution.

**Table 4:** Mean standard deviation and mean differences of all the variables measured between pre-test and post-test sessions

Variable	Experimental group (n = 33)			Control group (n = 18)		
	Pre-test	Post-test	Paired differences	Pre-test	Post-test	Paired differences
	M (SD)	M (SD)	M (SD)	M (SD)	M (SD)	M (SD)
Trust	65.58 (7.84)	74.48 (10.16)	$-8.91$ (9.99)	67.78 (7.12)	76.41 (9.13)	$-8.63$ (11.47)
Anthropomorphism	2.67 (0.64)	3.28 (0.66)	$-0.61$ (0.72)	2.78 (0.85)	2.98 (0.77)	$-0.20$ (1.03)
Animacy	3.20 (0.59)	3.76 (0.51)	$-0.56$ (0.68)	3.46 (0.67)	3.51 (0.52)	$-0.05$ (0.83)
Likeability	4.07 (0.56)	4.29 (0.62)	$-0.22$ (0.68)	4.03 (0.58)	4.10 (0.70)	$-0.07$ (0.71)
Perceived intelligence	3.89 (0.65)	4.18 (0.60)	$-0.28$ (0.59)	4.16 (0.65)	4.02 (0.64)	0.13 (0.80)
Perceived safety	3.91 (0.69)	3.97 (0.60)	$-0.06$ (0.84)	3.98 (0.79)	3.98 (0.60)	0.00 (0.73)

**Table 5:** Repeated measures ANOVA and ANCOVA results

Variable	ANOVAs									ANCOVAs		
	Group			Time			Time $\times$ Group			Self-talk		
	F(1, 48)	*p*	$\eta$	F(1, 48)	*p*	$\eta ^2$	F(1, 48)	*p*	$\eta ^2$	F(1, 48)	*p*	$\eta ^2$
Trust	0.92	0.34	0.02	$5.38^*$	0.03	0.10	0.01	0.94	0.00	0.19	0.66	0.00
Anthropomorphism	0.34	0.57	0.01	3.55	0.07	0.07	2.59	0.11	0.05	0.69	0.41	0.01
Animacy	0.00	0.99	0.00	1.39	0.24	0.03	$5.48^*$	0.02	0.10	0.07	0.80	0.00
Likeability	0.53	0.47	0.01	0.01	0.95	0.00	0.63	0.43	0.01	0.20	0.66	0.00
Perceived intelligence	0.15	0.70	0.00	0.23	0.63	0.01	$4.61^*$	0.04	0.09	0.62	0.43	0.01
Perceived safety	0.07	0.80	0.00	0.05	0.83	0.00	0.06	0.81	0.00	0.02	0.89	0.00

$$^{*}$$*p* 0.05

Tables 4 presents experimental and control groups descriptive statistics of all the variables measured between pre-test and post-test and Table 5 report the results of 2 $\times$ 2 factorial ANOVAs and ANCOVAs with repeated measures, performed on scores at the Trust and GODSPED questionnaires (anthropomorphism, animacy, likeability, perceived intelligence, perceived safety) collected during pre-test and post-test phases from both groups. Both factors Group and Time had two levels (Group: experimental and control; Time: pre-test, post-test).

In ANCOVAs, individuals’ score on self-talk questionnaire were used as covariate in order to examine to what extent the participants’ everyday use of self-talk influenced the effect of robot inner speech on trust.

**Fig. 6:**
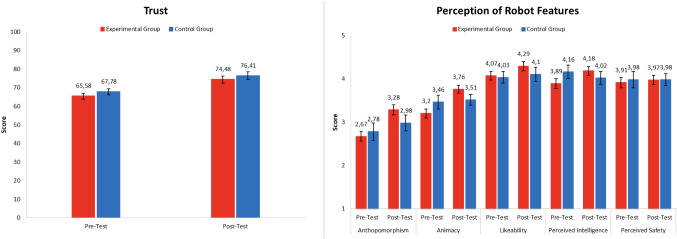
Scores of experimental and control group for all variables measured in pre-test and post-test sessions

Figure 6 reports graphic representation of group differences in pre- and post-test sessions.

The results of ANOVAs did not reveal a significant Group effect for trust [F(1, 48) = 0.92, *p* = 0.34, $\eta ^2$ = 0.02] indicating that there are no differences in both groups mean scores. On the contrary, a effect of Time for trust was found [F(1, 48) = 5.38, *p* < 0.05, $\eta ^2$ = 0.10] but not for the interaction Time $\times$ Group [F(1, 48) = 0.01, *p* = 0.94, $\eta ^2$ = 0.00].

These results indicate that all participants in both groups have improved their trust in the robot, from pre-test to post- test sessions, but that there are no differences in experimental and control group in the size of this effect. ANCOVA revealed also that participants’ rate of everyday self-talk has no influence on the effect of robot inner speech on trust [F(1, 48) = 0.19, *p* = 0.66, $\eta ^2$ = 0.00].

Concerning the different dimensions of users’ robot perception, results of the ANOVAs did not show a significant Group effect for anthropomorphism [F(1, 48) = 0.34, *p* = 0.57, $\eta ^2$ = 0.01], animacy [F(1, 48) = 0.00, *p* = 0.99, $\eta ^2$ = 0.00], likeability [F(1, 48) = 0.53, *p* = 0.47, $\eta ^2$ = 0.01], perceived intelligence [F(1, 48) = 0.15, *p* = 0.70, $\eta ^2$ = 0.00], and perceived safety [F(1, 48) = 0.07, *p* = 0.24, $\eta ^2$ = 0.00], indicating that there are no differences in both groups mean scores.

Also, no significant effect of Time was found [anthropomorphism: F(1, 48) = 3.55, *p* =0.07, $\eta ^2$ = 0.07; animacy: F(1, 48) = 1.39, *p* = 0.24, $\eta ^2$ = 0.03; likability: F(1, 48) = 0.01, *p* = 0.95, $\eta ^2$ = 0.00; perceived intelligence: F(1, 48) = 0.23, *p* =0.63, $\eta ^2$ = 0.01; perceived safety: F(1, 48) = 0.05, *p* = 0.83, $\eta ^2$ = 0.00].

However, a significant interaction effect Time $\times$ Group was found for the dimensions of animacy [F(1, 48) = 5.48, *p* < 0.05, $\eta ^2$ = 0.10] and perceived intelligence [F(1, 48) = 4.61, *p* < 0.05 $\eta ^2$ = 0.09]. These results indicate that means score of participants for the dimensions of animacy and perceived intelligence in the experimental group significantly improved compared to the one’s of participants in the control group from the pre-test to post-test sessions. In addition, for the perception of robot intelligence mean score of participants in the in the control group decrease between the two testing sessions. Concerning the others dimensions we found no statistically significant interaction effect of Time $\times$ Group [anthropomorphism: F(1, 48) = 2.59, *p* = 0.11, $\eta ^2$ = 0.05; likability: F(1, 48) = 0.63, *p* = 0.43, $\eta ^2$ = 0.01; perceived safety: F(1, 48) = 0.06, *p* = 0.81, $\eta ^2$ = 0.00], indicating that there was no significant mean difference between experimental and control groups from the pre-test to the post-test sessions.

ANCOVA revealed also that participants’ rate of everyday self-talk has no influence on the effect of robot inner speech on robot perception [anthropomorphism: F(1, 48) = 0.69, *p* =0.41, $\eta ^2$ = 0.01; animacy: F(1, 48) = $0-07$, *p* =0.80, $\eta ^2$ = 0.00; likability: F(1, 48) = 0.20, *p* = 0.66, $\eta ^2$ = 0.00; perceived intelligence: F(1, 48) = 0.62, *p* = 0.43, $\eta ^2$ = 0.01; perceived safety: F(1, 48) = 0.02, *p* = 0.89, $\eta ^2$ = 0.00].

## Discussions

This research aimed to investigate if the interaction with a robot equipped with an inner speech system during the execution of a cooperative task improves human trust levels and perception of robot anthropomorphic features. In addition, it was investigated the possible influence of human use of everyday self-talk on the perception of robot’s inner speech.

Concerning Trust, the results demonstrated that all participants’ trust scores significantly improved from pre-test to post-test, demonstrating that the interaction with the robot produced an increase in their trust levels. However, no Group x Time differences were found, indicating that the use of inner speech did not specifically influence the level of Trust toward robot in participants in the experimental group.

since the participants had never met face to face with a social robot before, it is possible to attribute this result to a sort of “novelty effects”; the simple interaction with a human-like robot increased trust in participants that is kind of robots before. That is consistent with studies [45, 46] demonstrating that trust is also shaped by history-based interaction: interaction with the robot changes the way human perceive and trust the robot, and this is particularly true in HRI with social robots that, like Pepper, look and behave like humans [30–38].

On the contrary, the results of users’ perception of robot revealed that only participants in the experimental group, who interacted with the robot equipped with inner speech, improved their perception of robots’ animacy and perceived intelligence from pretest to post-test, while there were not pre-/post-test differences in the control group. Even in this case, results were not influenced by individuals’ use of self-talk.

These results confirmed our hypothesis and support those studies that show that robot Pepper exhibiting human-like behaviors [30, 35, 45] are perceived as livelier and more intelligent than robot Pepper not showing human-like behaviors. In our experiment, through the overt inner speech system Pepper share with participants its thoughts and emotions, often addressing ironic and sarcastic comments to users. This particular interaction, by evidence, led users to perceive Pepper as more animated and intelligent. It is also possible that the ability of the robot to openly speak its mind made it easier for participants to understand its behaviors by forming a sort of mental representation of the robot. We found no effect of individuals’ use of inner speech on examined variable, indicating that the personal use of inner speech by participants in everyday situation did not influence the interaction with a robot equipped with inner speech system.

## Conclusions and Future Works

In conclusion, or study allowed to obtain two main findings. Firstly, they support the idea that, in social HRI, the more a robot shows human-like functioning the greater are humans perceptions about. A robot equipped with an inner speech system, which express his “thoughts”and explain its behaviors through an overt self-talk, is perceived animated and intelligent.

Secondarily, interaction with social robots, independently of the use of inner speech systems, increases trust in all participants to the experiment. Thus, in this case, inner speech does not play a specific role in improving users’ trust. This result may be due to different reasons, as follows: (1) involvement of novice participant: as already claimed, all participants were at the first interaction with Pepper, and the general novice effect of this first experience could have overcame and reduced the perception of the slight differences between the Inner speech/no inner speech conditions; (2) type of interaction: the proposed task did not represent an at risk situation for participants.

In the future, a new task integrating competitive environment together with cooperative one, could probably explicitly elicit more trustworthy towards robots. On the other hand, to the best of our knowledge, this is the first study to attempt at investigating if humans can trust more a robot that show, although rudimentary, inner speech. Future studies may allow to study further the effects of this new and robot feature.

## Data Availability

All data generated and/or analyzed during this study are available from the corresponding author on reasonable request.

## Footnotes

1 http://doc.aldebaran.com/2-5/naoqi/index.html.
